# The Scottish Universities

**Published:** 1903-09-05

**Authors:** 


### The Scottish Universities.

To those entrants upon the study of medicine who desire to obtain a university degree, and they are an increasing number, the Scottish Universities offer many attractions. They can show long and honourable traditions of efficiency and success. The college fees and other expenses are certainly not excessive, and can, indeed, be kept within wonderfully narrow limits by anyone content to cultivate medicine "on a little oatmeal." For the diligent and competent there is quite a respectable list of bursaries and scholarships. The Carnegie Trust will offer helping hands ready with gifts when Scottish blood is associated with *res angusta domi*. And the holder of a Scottish degree may be sure of finding many fellow graduates in whatever part of the world he may elect to practise. The weak point is the scarcity of attractive positions for the younger men. Fellowships and such like positions are few in number and carry no definite prospect of promotion. The salaries attached to lectureships, demonstratorships, etc., are small, and what is worse, these appointments afford no reasonable guarantee that they will lead to a professor's chair. Service in a Scottish University is therefore service in an organisation in which promotion is neither rapid nor certain. Hence it is not surprising that the Universities fail to keep many of their most promising graduates. The young graduate with brains and energy is disposed, not unnaturally, to take his talents to a sphere where the chances and rewards are more numerous and certain than those offered by his alma mater. Probably the Carnegie Trustees, by the foundation of Research Fellowships and other endowed appointments, may to some extent remedy this state of matters, but in the meantime the newly fledged graduate will be well advised to think twice before he stakes his fortunes on the chances of service among the junior appointments in a Scottish University.

It is almost impossible to overestimate the value of the influence and service rendered to the Scottish people by their national Universities. They cannot, it is true, claim the social distinction and training of Oxford and Cambridge. Nor have their funds permitted them to become nursing schools for the development of the higher types of scholarship. But they have done much to inspire and to gratify an ambition for knowledge and love of learning among all classes of the people, and through long generations have shown themselves quick to appreciate popular aspirations and demands. In these circumstances it is not surprising to find that the Universities have developed within their organisation elaborate training schools for practical life. Their Arts faculties are without question influential, and even predominant, but side by side with these there have grown up more specialised faculties devoted to instruction in such subjects as Divinity, Medicine, Law, and, more recently, Engineering. The theory has been to raise the teaching of these subjects to a university level, to make them instruments of a liberal education, and not merely to fit the graduate to make his livelihood by the practice of a technical art. This policy has perhaps not been particularly favourable to the development of the culture and scholarship which find their most congenial atmosphere away from the busy haunts of men and the sounds of commercial rivalry and strife. But it has had great advantages. One of these is that the more technical faculties have necessarily been influenced by mutual association, and have all felt the claim arising from a recognised university status. The professed aim of the teaching is not solely to confer expert knowledge and manipulative dexterity in the practice of a particular profession, but to develop the point of view from which men may "see life steadily and see it whole." The entire atmosphere of university life tells in the same direction. The environment for the medical student is therefore not that merely of a hospital and other medical students. On the contrary, it includes the liberalising influences supplied by large bodies of students who cultivate ambitions in many other directions than medicine, and these, in the nature of things, tend to develop breadth of view and accurate perspective. In short, the student who wishes to train himself to play a worthy part in the world and not merely to achieve a qualifying diploma will find in Scottish University life those opportune occasions which can hardly exist outside the circle of a large academic organisation.

